# THE MATCH/MISMATCH IN PERCEPTIONS OF SUCCESSFUL AGING AMONG OLDER BURUNDIAN REFUGEES IN THE US

**DOI:** 10.1093/geroni/igad104.1161

**Published:** 2023-12-21

**Authors:** Jonix Owino, Heather Fuller

**Affiliations:** Sacred Heart University, Stratford, Connecticut, United States; North Dakota State University, Fargo, North Dakota, United States

## Abstract

Perceptions of successful aging may be impacted by unique lived experiences such as migration, adaptation, and traumatic life experiences. While there exist studies on successful aging among immigrant communities, research studies on successful aging among older refugees, who flee due to life threatening situations, are sparse. The present qualitative study aimed to shed light on the match or mismatch that may exist between older refugees’ perceptions of successful aging and their perceptions of their lived experiences, as well as sex variations in those perceptions. Twenty-one Burundian refugees, aged 50+, were recruited from an upper Midwest community to participate in in-depth interviews. The qualitative interviews were analyzed using thematic content analysis. Themes denoting a match in aging refugees’ perceptions of success aging and lived experience included: wisdom/ability to give advice (i.e., guidance especially to younger individuals), experiencing peace and security, and fostering family relationships. A mismatch was highlighted in themes related to work experiences (i.e., the challenge of engaging in jobs not related to their prior skills), lack of social connections, and communication difficulties. Gender differences were apparent in that women emphasized shifts in work-related gender roles while men emphasized shifts in dependency-related gender roles. In many ways, aging refugees’ unique backgrounds and experiences seemed influential for their match/mismatch in successful aging perceptions and experiences. These findings highlight the importance for host communities to better understanding how refugees perceive and experience successful aging to best inform the implementation of effective interventions for aging refugees.

